# Supplementary material for: Enhanced Two-Photon Fluorescence and Fluorescence Imaging of Novel Probe for Calcium Ion by Self-Assembly with Conjugated Polymer
Source: Polymers (Basel). 2019 Oct 10;11(10):1643. doi: 10.3390/polym11101643 (PMC6835751; doi:10.3390/polym11101643)
Supplement: Supplementary file 1 [file polymers-11-01643-s001.pdf]

Supporting information

Enhanced two-photon fluorescence and fluorescence imaging of a novel probe for calcium ion by self-assembly with a conjugated polymer

Yue-liang Zhai [a, b, ‡], Qiu-bo Wang [a, b, ‡], Hao Yu [a, b], Xiao-yuan Ji [a, b], Xian Zhang* [a, b]

[a] School of Materials Science and Engineering，Qilu University of Technology (Shandong Academy of Sciences)，Jinan 250353，China

[b] Shandong Provincial Key Laboratory of Processing and Testing Technology of Glass and Functional Ceramics; Key Laboratory of Amorphous and Polycrystalline Materials; Qilu University of Technology, Jinan 250353, China

*Correspondence: zhangx@qlu.edu.cn; Tel.: +86-531-89631227

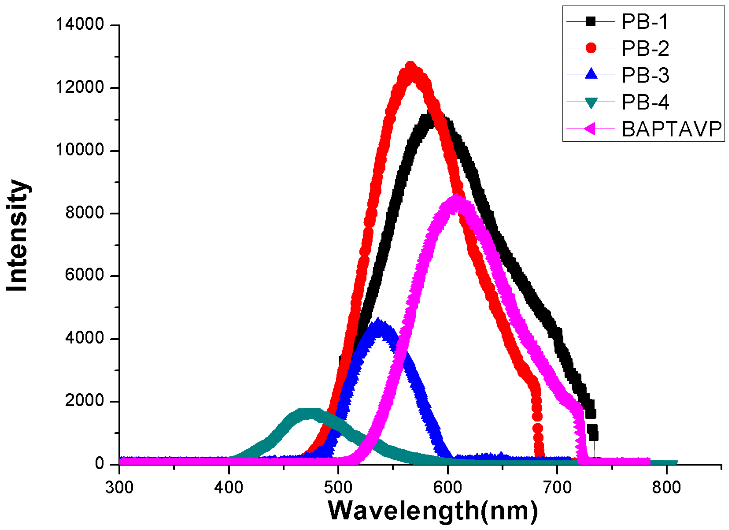

Fig. S1. The fluorescence spectra of PCBMB-BAPTAVP with the different concentrations at the excitation wavelength 800 nm. Probe: BAPTAVP; PB: PCBMB: BAPTAVP =1:1; PB-1: 5×10^{-4} mol/L; PB -2, 1×10^{-4} mol/L; PB -3: 5×10^{-5} mol/L; PB -4: 1×10^{-5} mol/L.

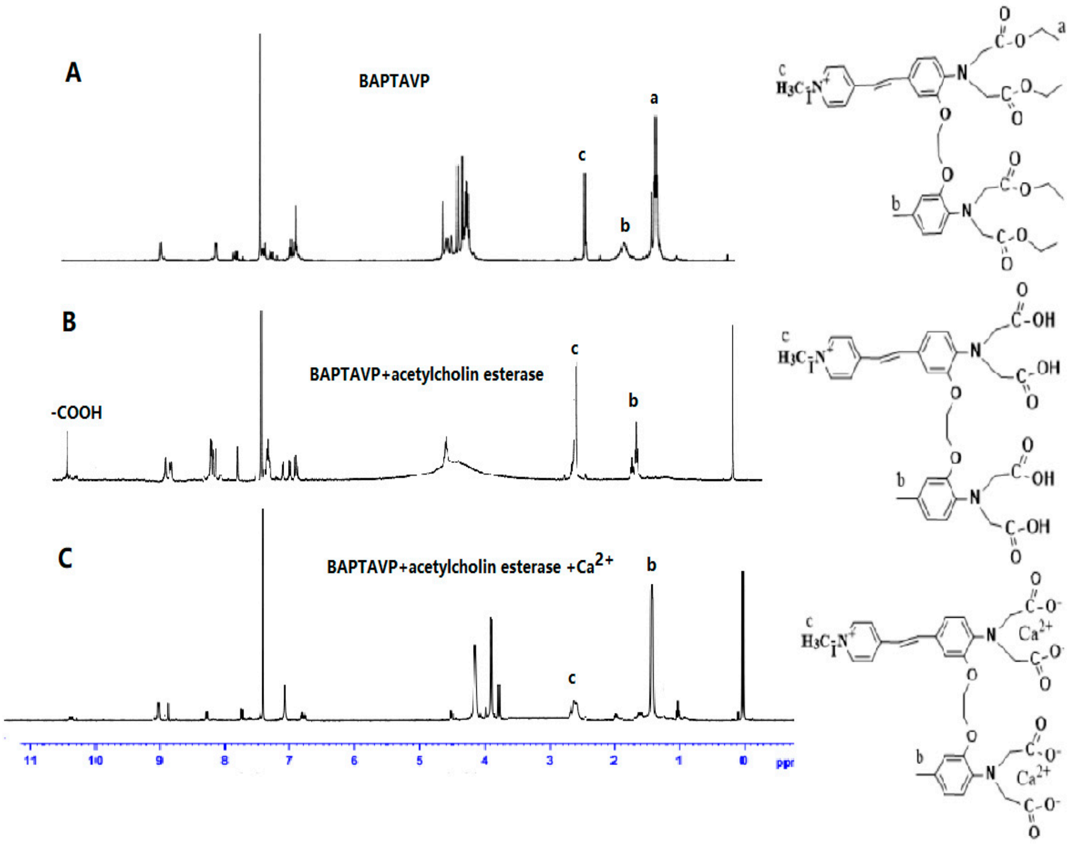

Fig. S2. The ^{1}H NMR spectra of BAPTAVP(A), BAPTAVP in the presence of acetylcholin esterase(B) and BAPTAVP in the presence of acetylcholin esterase and Ca^{2+}(C) in $CDCl_3$.
